# Atorvastatin downregulates co-inhibitory receptor expression by targeting Ras-activated mTOR signalling

**DOI:** 10.18632/oncotarget.21003

**Published:** 2017-09-18

**Authors:** Isobel Okoye, Afshin Namdar, Lai Xu, Nicole Crux, Shokrollah Elahi

**Affiliations:** ^1^ Department of Dentistry, Faculty of Medicine and Dentistry, University of Alberta, Edmonton, T6G 2E1 Canada; ^2^ Department of Medical Microbiology and Immunology, Faculty of Medicine and Dentistry, University of Alberta, Edmonton, T6G 2E1 Canada

**Keywords:** inhibitory receptors, atorvastatin, immune checkpoints

## Abstract

Regulation of T cell function in the steady state is mediated by co-inhibitory receptors or immune checkpoints such as PD-1, CTLA-4, TIM-3 and LAG-3. Persistent antigen stimulation, during chronic viral infections and cancer, results in sustained expression of multiple co-inhibitory receptors and subsequently poor effector T cell function. Immune checkpoint blockade using monoclonal antibodies against PD-1, PDL-1 and CTLA-4 has been implemented as an immunotherapy strategy- resulting in restoration of T cell function and reduction of viral load or tumour growth. Immunomodulatory roles of commonly used cholesterol-lowering medications, atorvastatin and other statins, are widely documented. We have previously shown that atorvastatin can inhibit HIV-1 infection and replication. Here, for the very first time we discovered that atorvastatin also regulates activated T cell function by mediating downregulation of multiple co-inhibitory receptors, which corresponded with increased IL-2 production by stimulated T cells. In addition, we found that atorvastatin treatment reduces expression of mTOR and downstream T cell effector genes. We demonstrate a novel mechanism showing that atorvastatin inhibition of Ras-activated MAPK and PI3K-Akt pathways, and subsequent mTOR signalling promotes gross downregulation of co-inhibitory receptors. Thus, our results suggest that statins may hold particular promise in reinvigorating T cell function in chronic conditions.

## INTRODUCTION

In chronic viral infections such as HIV, HCV and cancer, T cells receive persistent molecular signals from antigens or inflammation. This induces a state of dysfunctionality in T cells termed T cell exhaustion [1]. Exhausted T cells lose their robust effector functions such as the ability to produce cytokines, proliferative capacity, and eventually the ability to kill virus-infected cells and tumor cells. Instead they over-express multiple co-inhibitory receptors (immune checkpoints) [1, 2]. Transient expression of co-inhibitory receptors regulates T cell tolerance by restraining initial T cell activation that otherwise can be potentially pathogenic. However, increased and sustained expression of co-inhibitory receptors such as PD-1, CTLA-4, TIM-3 and LAG-3 are hallmarks of T cell exhaustion [3]. Co-inhibitory receptors upon interaction with their ligands on antigen presenting cells (APCs) and/or tumor cells mediate immune suppression [4]. Thus, blockade of co-inhibitory receptor-ligand interactions is an exciting novel approach to reinvigorate exhausted T cells and one of the main objectives of immunotherapy [5, 6]. Recent advances in the field have demonstrated that T cell exhaustion is reversible in several type of cancers and this has attracted significant attention as a novel game-changing immunotherapy strategy [7–9].

Statins are one of the main compounds that inhibit 3-hydroxy 3-methylglutaryl coenzyme A (HMG-CoA) reductase, the major enzyme in the cholesterol synthesis pathway [10, 11]. HMG-CoA reductase facilitates the conversion of HMG-CoA to L-mevalonate, which is the main metabolic precursor of cholesterol. In addition, L-mevalonate is the main metabolite in the production of the prenylated proteins, farnesyl pyrophosphate and geranyl geranyl pyrophosphate [12] and Supplementary Figure 1.

Due to their ability to block (HMG-CoA) reductase, statins are primarily used to treat hypercholesterolemia and prevent atherosclerosis [13]. Isoprenoids, the major product of L-mevalonate pathway, are essential for small GTPase activity, which are involved in cell signaling, cytotoxic T cell function and formation of the immunological synapse [14]. Therefore, in addition to their cholesterol-lowering properties, statins exert broad spectrum immunomodulatory and anti-inflammatory effects [15]. Of note, the anti-inflammatory function of statins is extensively linked to the activity of T cells [14]. Previous reports have revealed that statins, like simvastatin and pravastatin for instance, are able to reduce inflammatory T cell responses [16–18]. In experimental autoimmune encephalomyelitis (EAE), a mouse model of multiple sclerosis, switching from a pathogenic Th1 to a Th2 response was observed after administration of lovastatin or atorvastatin [19, 20]. Atorvastatin, one of the high potency statins [21], suppresses Fas ligand (FasL) expression and cytotoxicity due to inhibition of isoprenoid production [22, 23].

We have previously shown that atorvastatin inhibits HIV-1 replication in CD4^+^ T cells by facilitating upregulation of the cyclin-dependent kinase inhibitor p21 [24]. Other studies have also demonstrated the ability of atorvastatin and other statins to modulate T cell activation and function in HIV-1 infection [18, 25]. More importantly, it has been reported that atorvastatin reduces T cell exhaustion in HIV-1 infected patients on antiretroviral therapy [24].

Cholesterol is an essential part of mammalian cellular membranes. Highly proliferative cells such as cancer cells require more cholesterol to synthesize membranes quickly [26]. Statins, by reducing intracellular biosynthesis of cholesterol may influence tumour proliferation and exhibit anticancer activity. Although the epidemiological evidence about the role of statins in cancer patients are mixed [27, 28], multiple studies have shown a role for statins in reducing tumour development by targeting the L-mevalonate pathway [29] [26, 30]. For instance, an association between statin use and 15% reduction in cancer-related mortality has been reported [30]. In addition, a decreased risk of breast cancer recurrence in patients taking statin has been shown [31]. However, the potential role of statins on expression of co-inhibitory receptors has never been studies. As discussed above, PD-1 and other co-inhibitory receptors facilitate T cell exhaustion during chronic viral infections and cancers [1, 32, 33], [34]. Therefore in this study we investigated the immunomodulatory effects of atorvastatin, the most commonly prescribed statin [35] with the highest safety profile [36], on the expression of co-inhibitory receptors by using human peripheral blood mononuclear cells (PBMCs) *in vitro*. Here, we, for the very first time, demonstrate a novel role for atorvastatin in reducing the expression of co-inhibitory receptors (e.g. PD-1, CTLA-4, TIM-3, LAG-3, 2B4, TIGIT, CD160) and their ligands (e.g. PDL-1 and galectin-9 (Gal-9)). We found that atorvastatin can potentially target co-inhibitory receptors and thus may facilitate restoration of T cell responses in chronic conditions.

## RESULTS

### Targeting PD-1, CTLA-4 and PD-L1 by antibody blockade increases IL-2 production and T cell proliferation *in vitro*

**Figure 1:**
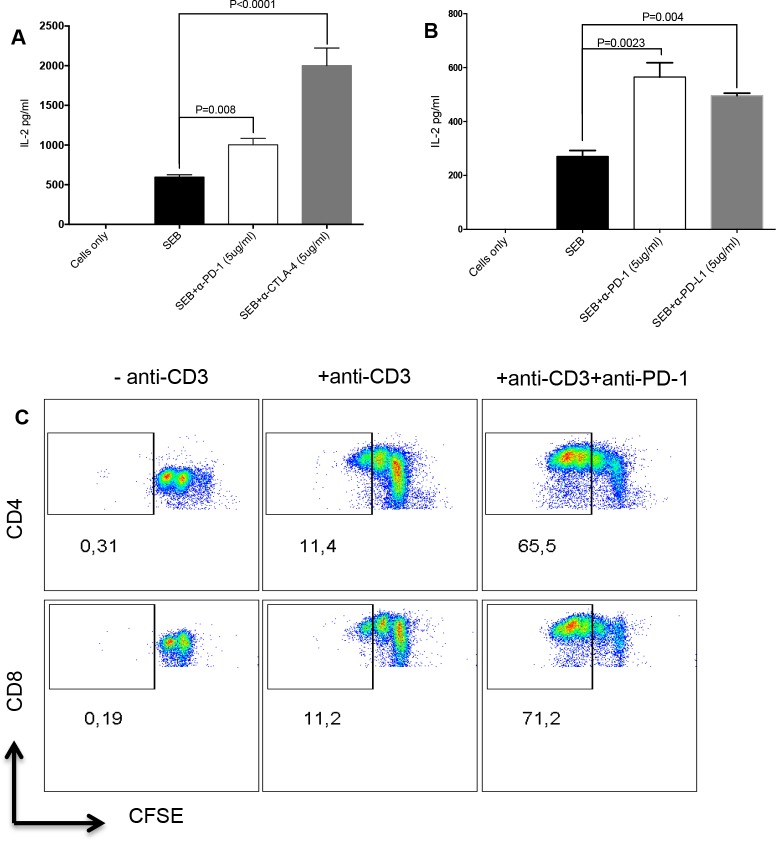
Targeting co-inhibitory receptors by antibody blockade increases T cell proliferation and IL-2 production **(A)** Bar graphs showing production of IL-2 in cell culture supernatants from SEB-stimulated cells treated with anti-PD-1 antibody (5 μg/ml) or anti-CTLA-4 (5 μg/ml) antibody for 72hrs. **(B)** IL-2 production by SEB-stimulated cells treated with anti-PD-1 (5 μg/ml) or anti-PD-L1 (5 μg/ml) for 72hrs. Unstimulated and stimulated-only cultures were used as controls. Data are representative of three independent experiments. Bar, mean ± one standard error. **(C)** Representative FACS plots showing CFSE dilution in CD4^+^ (top) and CD8^+^ T cells (bottom) stimulated with anti-CD3 with or without anti-PD-1 treatment for 72hrs. Unstimulated (-anti-CD3) or stimulated (+anti-CD3) cells were used as controls.

Blocking co-inhibitory receptors expressed by T cells using antibodies restores their cytokine-producing ability and proliferative capacity [6]. We utilized an *in vitro* assay to mimic T cell exhaustion. We show here that stimulation of PMBCs with Staphylococcal Enterotoxin B (SEB) or anti-CD3/CD28 upregulates expression of co-inhibitory receptors and blockade of some of these receptors enhances T cell functions. We found that SEB stimulation of PBMCs for 72hrs in the presence of α-PD-1, CTLA-4 or PD-L1 antibodies led to a significant increase in IL-2 production (Figure 1A, 1B). Similarly, we observed increased proliferation, depicted by Carboxyfluorescein succinimidyl ester (CFSE)-dilution, of CD4^+^ and CD8^+^ T cells stimulated with α-CD3/CD28 and treated with anti-PD-1 for 72hrs, compared to stimulated-only controls (Figure 1C). With these observations, we decided to use this *in vitro* system to investigate the effect of atorvastatin on co-inhibitory receptor expression and restoration of T cell function.

### Reduced expression of multiple co-inhibitory receptors by CD4^+^ and CD8^+^ T cells in the presence of atorvastatin

**Figure 2:**
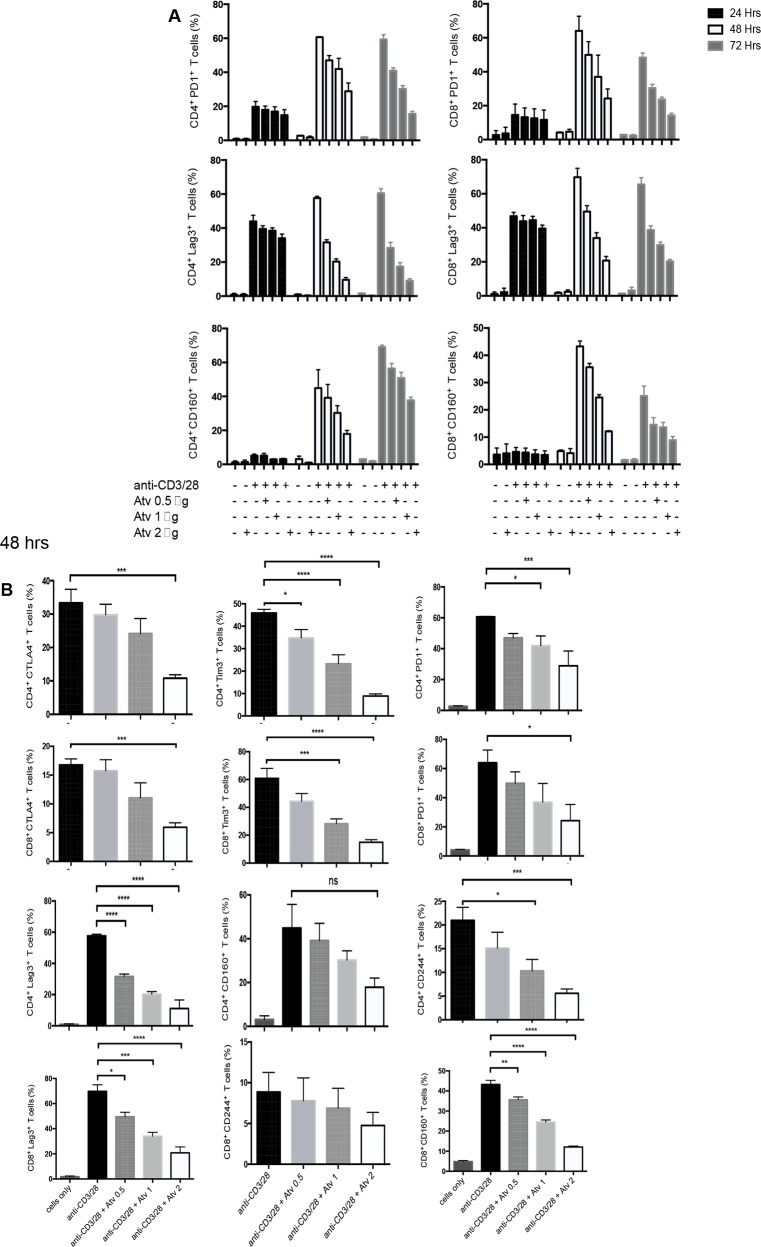
Stimulation of PBMCs by α-CD3/CD28 in the presence of atorvastatin results in reduced expression of co-inhibitory receptors by CD4+ and CD8+ T cells **(A)** Downregulation in co-inhibitory receptor expression by stimulated T cells in response to atorvastatin treatment occurs over time in a dose-dependent manner. Percentages of PD-1, LAG-3 and CD160 expressed by CD4^+^ (left) and CD8^+^ (right) T cells in response to α-CD3/CD28 stimulation in the presence of the indicated concentrations of atorvastatin after 24, 48 and 72hrs. Data from three independent experiments shown. **(B)** Bar graphs showing cumulative expression of PD1, LAG-3, TIM-3, CD160, CTLA-4 and CD244 by CD4^+^ and CD8^+^ T cells stimulated with α-CD3/CD28 with or without atorvastatin (1 μg/ml) for 48hrs. Unstimulated (cells only) cultures were used as negative controls. P values are defined by ^*^ (P ≤ 0.05) ^**^ (P < 0.01), ^***^ (P < 0.001) and ^****^ (P < 0.0001).

**Figure 3:**
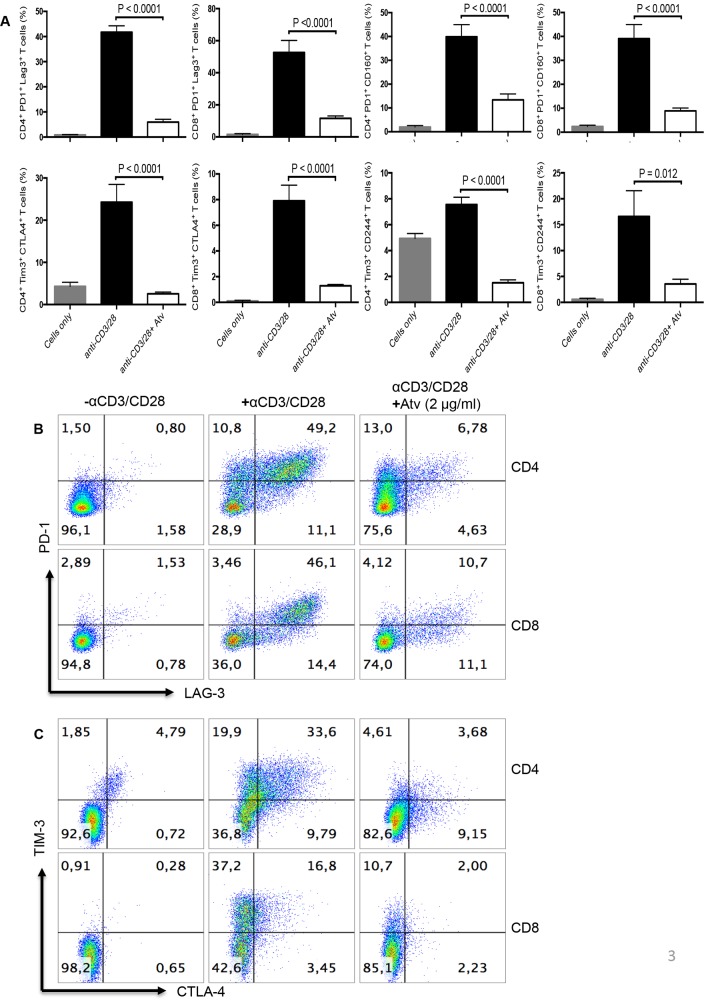
Stimulation of PBMCs by α-CD3/CD28 in the presence of atorvastatin results in reduced co-expression of co-inhibitory receptors by CD4+ and CD8+ T cells **(A)** Bar graphs showing co-expression of indicated co-inhibitory receptors by α-CD3/CD28-stimulated in the presence and absence of atorvastatin (1 μg/ml) (right panel) CD4^+^ and CD8^+^ T cells (48hrs). Data are representative of at least five independent experiments. Bar, mean ± one standard error. **(B)** Representative dot plots showing co-expression of PD-1 and LAG-3 and **(C)** CTLA-4 and TIM-3 by unstimulated (left panel), α-CD3/CD28-stimulated (middle panel) and stimulated plus atorvastatin (right panel) CD4^+^ and CD8^+^ T cells (48hrs). P values are defined by ^*^ (P ≤ 0.05) ^**^ (P < 0.01), ^***^ (P < 0.001) and ^****^ (P < 0.0001).

**Figure 4:**
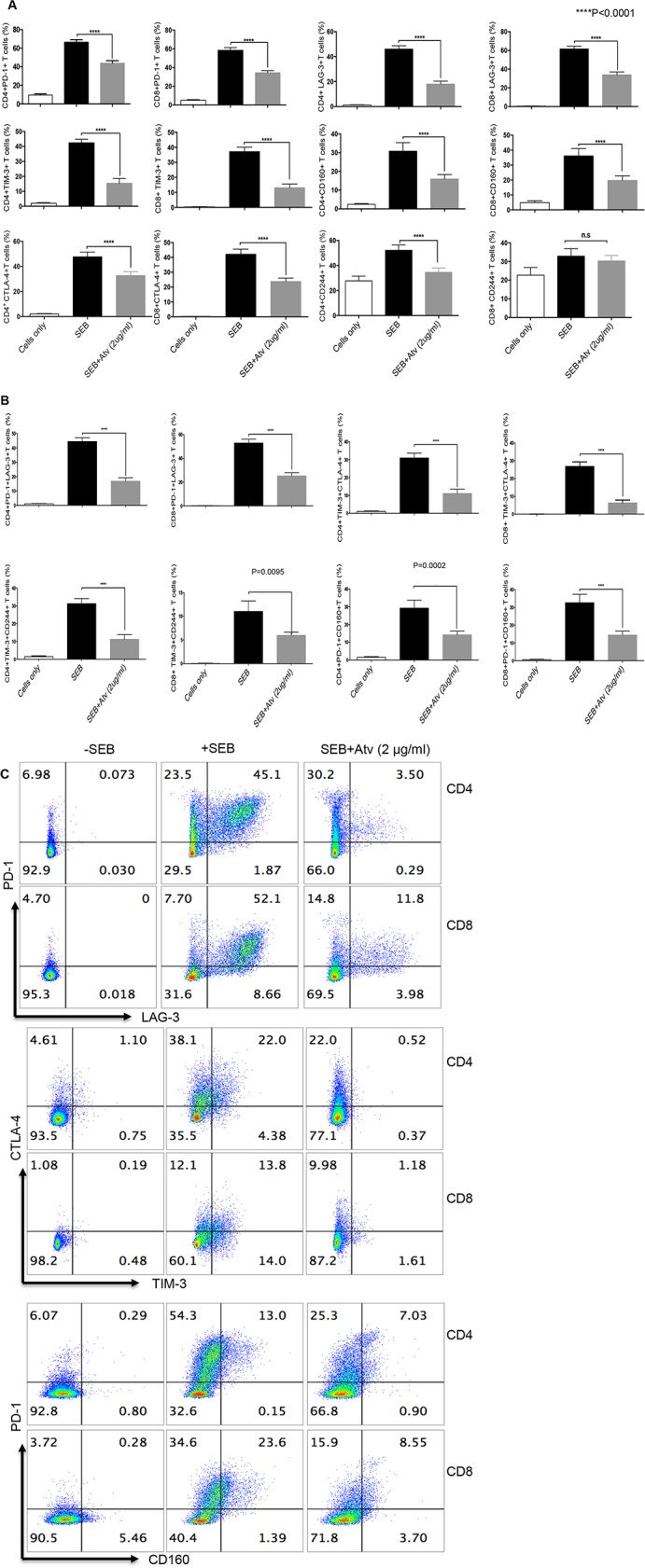
Expression of co-inhibitory receptors is reduced in CD4+ and CD8+ T cells stimulated with SEB in the presence of atorvastatin **(A)** Bar graphs showing expression of PD-1, LAG-3, TIM-3, CD160, CTLA-4 and CD244 by CD4^+^ and CD8^+^ T cells stimulated with SEB with or without atorvastatin (2 μg/ml) for 72hrs. **(B)** Co-expression of indicated co-inhibitory receptors in response to SEB-stimulation and atorvastatin treatment. **(C)** Representative dot plots indicating co-expression of co-inhibitory receptors in the presence and absence of atorvastatin. Results are representative of at least five independent experiments. Bar, mean ± one standard error. Unstimulated (cells only) cultures were used as negative controls.

In order to identify the effect of atorvastatin on T cells, PBMCs were stimulated using α-CD3/CD28 or SEB for 24, 48 or 72hrs with different concentrations of atorvastatin. In humans, 80 mg of atorvastatin daily is the highest recommended dose for treatment of hypercholesterolemia [36]; for an individual who weighs 60 kg this dose equals 1.4 mg/kg or ~1.4 μg/ml. Thus, for our studies we used physiologically relevant doses ranging from 20 to less than 80 mg/kg. Using these culture conditions, we consistently found that CD4^+^ and CD8^+^ T cells stimulated with α-CD3/CD28 in the presence of atorvastatin exhibited significant reduction in the expression of PD-1, LAG-3, CD160, TIM-3, CTLA-4 and 2B4 (CD244) in a dose-dependent manner after 48 and 72hrs (Figure 2A, 2B and Supplementary Figure 2A). Interestingly, 24hr treatment with atorvastatin had no significant effects on co-inhibitory receptor expression (data not shown), which is consistent with our previous reports on the modulatory effects of atorvastatin on HIV-1 replication in CD4^+^ T cells [24]. Since 48 and 72 hr treatments induced significant reduction in co-inhibitory receptor expression without compromising cell viability (Supplementary Figure 2B) therefore, 48 and/or 72 hr atorvastatin treatment was used in subsequent experiments. As shown in Figure 2A, 2B, Figure 3A–3C and Supplementary Figure 2A, the percentages of T cells expressing co-inhibitory receptors significantly reduced when treated with the higher concentrations of atorvastatin (1-2 μg/ml) compared to stimulated-only controls. In some cases, even 0.5 μg/ml atorvastatin significantly reduced the expression of co-inhibitory receptors (e.g. CD160, LAG-3, Tim-3 and PD-1) (Figure 2B and Supplementary Figure 2A). In contrast, despite some reduction in expression of 2B4 on CD8^+^ T cells following treatment with atorvastatin these changes were not significant (Figure 2B). Of note, it appears that stimulation with α-CD3/CD28 results in lower expression of CD160 on CD8^+^ T cells compared with CD4^+^ cells after 72hrs (Figure 2A). A similar pattern of significant co-inhibitory receptor downregulation was observed in atorvastatin-treated, SEB-stimulated CD4^+^ and CD8^+^ T cells after 72hrs (Figure 4A, Supplementary Figure 3A). Furthermore, the frequencies of co-inhibitory receptors co-expressed by CD4^+^ and CD8^+^ T cells were reduced following treatment with atorvastatin compared to untreated controls (Figure 4B, 4C, Supplementary Figure 3B). In addition to downregulation of various co-inhibitory receptors, we found that atorvastatin-treated and stimulated CD4^+^ and CD8^+^ T cells expressed reduced levels of the activation marker CD71 (Supplementary Figure 3C, 3D). These results indicate that atorvastatin modulates T cell function by facilitating the downregulation of activation markers, including multiple co-inhibitory receptors.

### Expression of co-inhibitory receptor ligands is reduced in response to atorvastatin treatment

**Figure 5:**
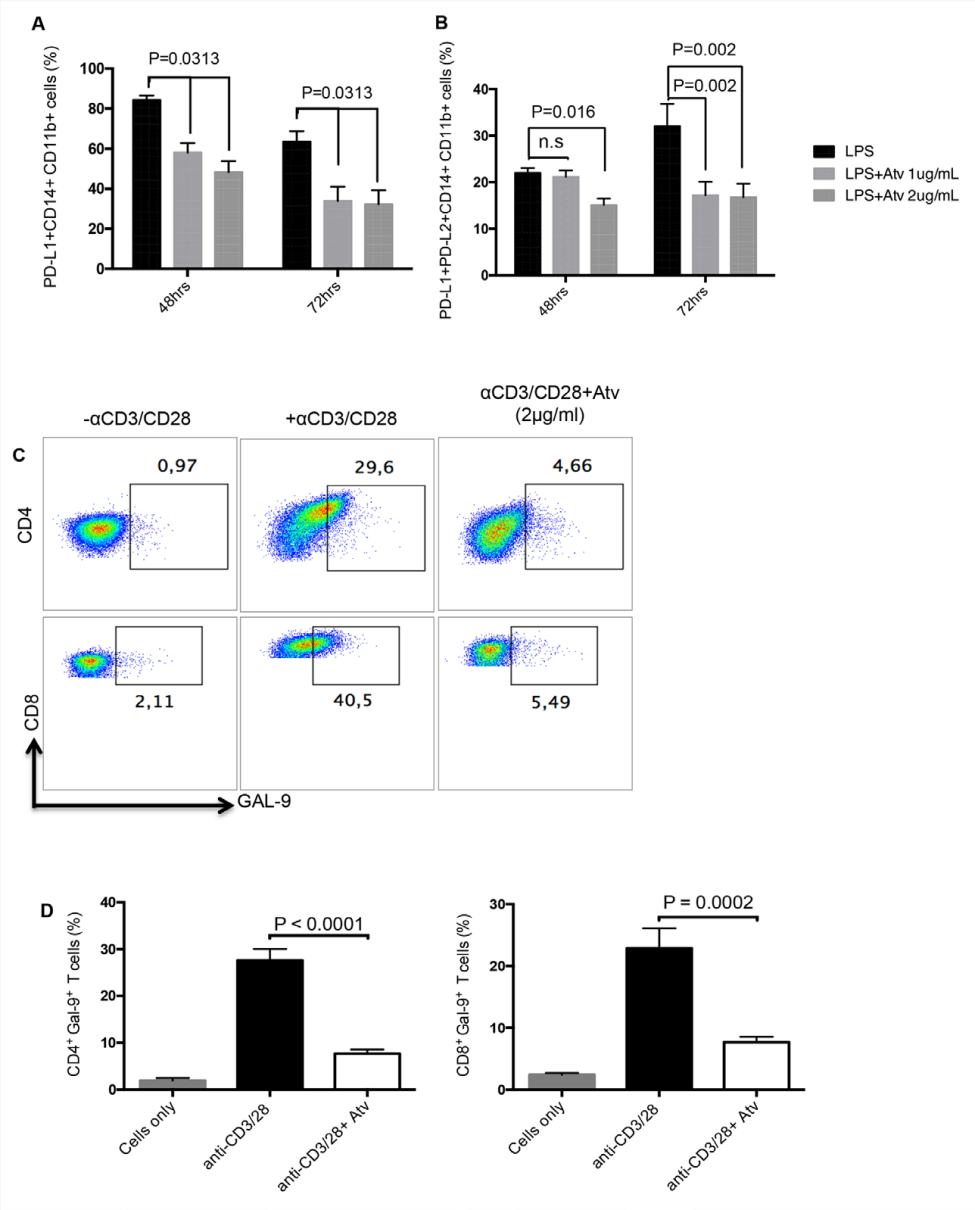
Expression of co-inhibitory receptor ligands are reduced in response to atorvastatin treatment **(A)** Bar graphs showing expression of PD-L1 by CD11b^+^CD14^+^ monocytes from LPS-stimulated cultures in the presence of atorvastatin (1 or 2 μg/ml) for 48 and 72hrs. **(B)** Bar graphs showing co-expression of PD-L1 and PD-L2 by CD11b^+^CD14^+^ monocytes from LPS-stimulated and atorvastatin-treated (1 or 2μg/ml) cultures at 48 and 72hrs. **(C)** Representative dot plots showing expression of Gal-9 by CD4^+^ or CD8^+^ T cells from α-CD3/CD28-stimulated cultures alone, or in the presence of atorvastatin (2 μg/ml) for 48hrs. **(D)** Bar graphs showing expression of Gal-9 by CD4^+^ (right) and CD8^+^ (left) T cells from α-CD3/CD28-stimulated cultures, with or without atorvastatin (2 μg/ml). Results are representative of three independent experiments. Bar, mean ± one standard error.

Based on the observation that atorvastatin facilitates downregulation of co-inhibitory receptors expressed by CD4^+^ and CD8^+^ T cells, we hypothesised that expression of co-inhibitory receptor ligands will be affected by this treatment. We thereby investigated the expression of the PD-1 ligands, PD-L1 and PD-L2, on CD11b^+^ CD14^+^ monocytes stimulated with LPS in the presence of atorvastatin (1-2 μg/ml). Similar to PD-1, we observed a significant reduction in the percentage of PD-L1- expressing monocytes following stimulation in the presence of atorvastatin at 48 and 72hrs compared to controls (Figure 5A). Furthermore, the percentage of stimulated monocytes that co-expressed PD-L1 and PD-L2 was reduced in response to atorvastatin treatment at both time-points studied (Figure 5B). In addition we found that expression of the TIM-3 ligand, Gal-9 on CD4^+^ and CD8^+^ T cells following stimulation with α-CD3/CD28 (48hrs) was significantly reduced in the presence of atorvastatin (Figure 5C, 5D). Similarly reduction of Gal-9 expression by SEB-stimulated and atorvastatin-treated CD4^+^ and CD8^+^ T cells was observed (data not shown). Thus, atorvastatin can modulate the expression of co-inhibitory receptors and their ligands.

### The effects of atorvastatin on CD4^+^ and CD8^+^ T cell proliferation and cytokine production

**Figure 6:**
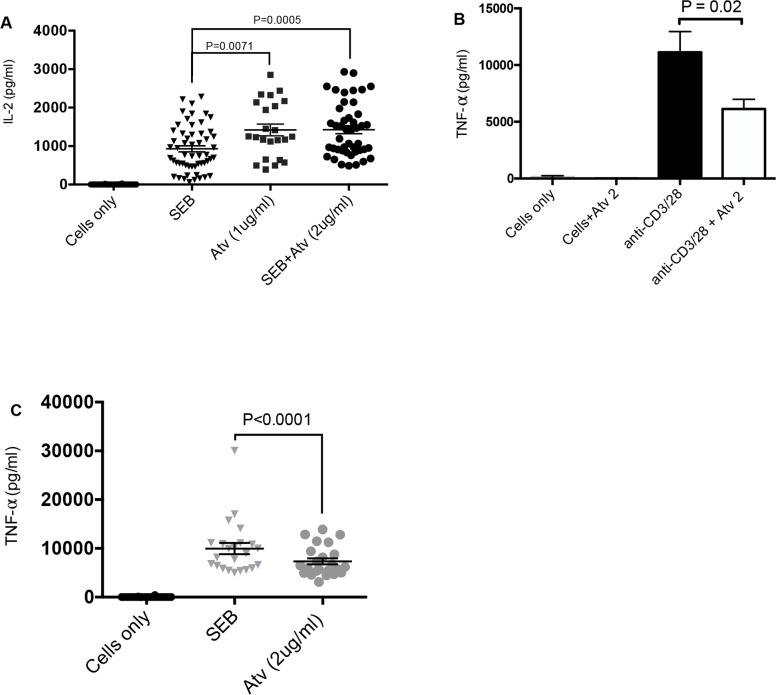
Cytokine production by stimulated PBMCs in response to atorvastatin treatment **(A)** Scatter plots with bars showing concentration of IL-2 in cell culture supernatants from unstimulated, SEB-stimulated and stimulated PBMCs in the presence and/or absence of atorvastatin (1 or 2 μg/ml). **(B)** and **(C)** Bar graph and scatter plot showing concentration of TNF-α in cell culture supernatants stimulated by α-CD3/CD28 and SEB with or without atorvastatin treatment. Bar, mean ± one standard error. Each point represents data from an individual PBMC, representative of more than three independent experiments.

Next we investigated whether reduction in co-inhibitory receptor expression in the presence of atorvastatin enhances the functionality of CD4^+^ and CD8^+^ T cells in terms of proliferation and cytokine production. Interestingly, proliferation of CD4^+^ and CD8^+^ T cells in response to both α-CD3/CD28 and SEB stimulation was hampered in the presence of atorvastatin (data not shown). However, we observed significantly higher IL-2 production in response to SEB stimulation (72hrs) in the presence of atorvastatin (Figure 6A). We also investigated the effect of atorvastatin treatment on the production of IFN-γ and TNF-α. Production of IFN-γ was not affected by atorvastatin treatment (data not shown); however, the concentrations of TNF-α in culture supernatants of stimulated and atorvastatin-treated cells (α-CD3/CD28 and SEB stimulated) were considerably lower compared to untreated controls (Figure 6B, 6C). Of note, at lower concentrations (0.5 and 1 μg/ml) of atorvastatin TNF-α production was unchanged (data not shown). Thus, atorvastatin treatment compromises T cell proliferation and reduces TNF-α production but enhances IL-2 production by SEB stimulated T cells.

### Atorvastatin mediates downregulation of co-inhibitory receptors through the L-mevalonate pathway

**Figure 7:**
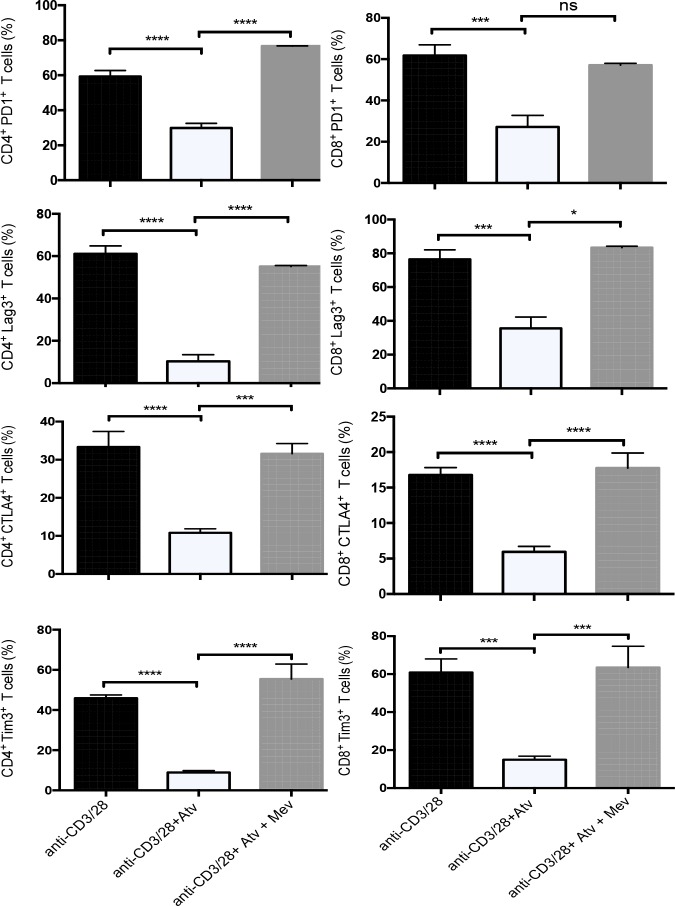
Downregulation of co-inhibitory receptors by atorvastatin is reversed by L-mevalonate Bar plots showing percentage of co-inhibitory receptors expressed by CD4^+^ and CD8^+^ T cells following α-CD3/CD28-stimulation (48hrs) in the presence and absence of atorvastatin and/or L-mevalonate (100μM) compared to controls. Data from three independent experiments. Bar, mean ± one standard error. P values are defined by ^*^ (P ≤ 0.05), ^***^ (P < 0.001) and ^****^ (P < 0.0001).

We hypothesized that the observed effects of atorvastatin on decreasing co-inhibitory receptors were mediated by inhibiting HMG-CoA reductase. Activated T cells were treated with atorvastatin alone or atorvastatin plus exogenous L-mevalonate, the product of HMG-CoA reductase. The addition of L-mevalonate to α-CD3/CD28- and atorvastatin-treated cultures resulted in increased expression of PD-1, LAG3, TIM-3, CTLA-4 and CD160 by CD4^+^ and CD8^+^ T cells, thereby reversing the effects of atorvastatin (Figure 7). Similar reversal effects of atorvastatin were observed when PBMCs were stimulated with SEB in the presence of L-mevalonate (data not shown). However, L-mevalonate alone had no significant effects on the expression of co-inhibitory receptors.

We also examined cytokine production by stimulated cells in response to addition of L-mevalonate. The concentration of IL-2 produced in response to atorvastatin plus L-mevalonate was lower than atorvastatin only cultures and comparable to SEB-stimulated only cultures; however this did not reach statistical significance (Supplementary Figure 4A). Also, L-mevalonate treatment reversed the pattern of TNF-α production by α-CD3/CD28-stimulated cells (Supplementary Figure 4B). These results indicate that addition of L-mevalonate can reverse atorvastatin-induced changes in the phenotype and function of stimulated CD4^+^ and CD8^+^ T cells.

We also investigated whether addition of exogenous farnesyl, a downstream product of the L-mevalonate pathway, can reverse atorvastatin-mediated co-inhibitory receptor downregulation. The pattern of co-inhibitory receptor expression by α-CD3/CD28-stimulated and atorvastatin-treated CD4^+^ and CD8^+^ T cells was reversed by farnesyl. However, this was significant for some of co-inhibitory receptors analysed (Supplementary Figure 4C).

As statins facilitate inhibition of cholesterol production, we examined if addition of cholesterol to cultures impact co-inhibitory receptor expression. Interestingly, the addition of cholesterol during PBMC stimulation did not produce any significant changes in co-inhibitory receptor expression and IL-2 production by CD4^+^ and CD8^+^ T cells with or without atorvastatin treatment (Supplementary Figure 5A, 5B). Thus, L-mevalonate, but not farnesyl could significantly restore atorvastatin-induced changes in co-inhibitory receptor expression and IL-2 production by CD4^+^ and CD8^+^ T cells. The addition of cholesterol however, has no impact on the expression of co-inhibitory receptors by T cells treated with atorvastatin.

### Atorvastatin reduces the expression of mechanistic target of rapamycin (mTOR) and the transcription factors T-bet, GATA3, BATF and FOXO1 by stimulated CD4^+^ and CD8^+^ T cells

**Figure 8:**
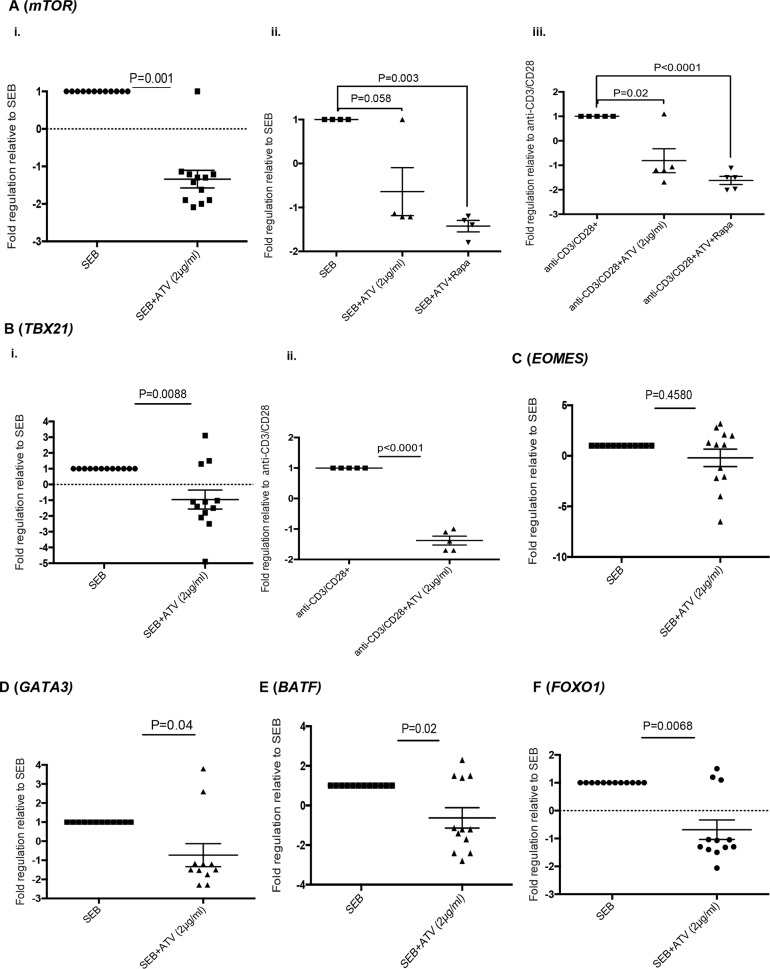
Atorvastatin-treated T cells express reduced levels of mTOR, T-bet, GATA3, BATF and FOXO1 Scatter plots showing expression of indicated genes in response to stimulation with or without atorvastatin treatment. **(A)** Expression of mTOR by SEB-stimulated T cells (72hrs) in response to atorvastatin treatment (i), atorvastatin with rapamycin (ii) and by α-CD3/CD28-stimulated T cells in response to atorvastatin treatment with or without rapamycin for 48hrs. **(B)** Expression of T-bet (*TBX21*) by SEB-stimulated (i) and α-CD3/CD28-stimulated T cells (ii), with or without atorvastatin treatment for 72 and 48hrs respectively. **(C-F)** Expression of EOMES, GATA3, BATF and FOXO1 by T cells stimulated with SEB with or without atorvastatin respectively. The fold regulation of indicated genes relative to stimulated-only controls is shown. Data are from 3 independent experiments, not significant (n.s). Bar, mean ± one standard error.

Atorvastatin and other statins inhibit farneslyation-dependent, Ras-activated pathways such as MAPK signalling and the PI3K-AKt-mTOR pathway [14]. In this regard, we investigated the expression of mTOR by stimulated T cells in response to atorvastatin treatment. mTOR mRNA levels in T cells stimulated with SEB and α-CD3/CD28 in the presence of atorvastatin was significantly reduced than stimulated-only controls (Figure 8A, i, ii, iii). As expected, mTOR expression levels by T cells isolated from rapamycin-treated cultures were lower than untreated controls (Figure 8A, ii, iii).

mTOR induction by TCR signalling is essential for effector T cell function mediated by transcription factors such as T-bet (*TBX21*), GATA3, Eomesodermin (*EOMES*) and BATF [37–39]. We therefore investigated whether expression of these genes were affected by atorvastatin treatment. mRNA levels of T-bet, GATA3 and BATF were significantly lower in T cells stimulated by SEB in the presence of atorvastatin (Figure 8B(i), 8D, 8E). We also observed a similar pattern in T-bet expression by atorvastatin-treated, α-CD3/CD28-stimulated T cells (Figure 8B(ii)). However, we found no difference in *EOMES* expression between atorvastatin treated and untreated SEB-stimulated T cells (Figure 8C). The activity of the transcription factor FOXO1 has been shown to increase upon mTOR inhibition during chronic viral infections and increased expression of co-inhibitory receptors such as PD-1 [40]. The presence of atorvastatin during SEB stimulation however resulted in reduced expression of FOXO1 by responding T cells (Figure 8F).

These observations infer that atorvastatin can utilise a multi-pronged approach to counter T cell activation and effector cell differentiation and function. In addition to targeting proximal co-inhibitory receptors, atorvastatin inhibits expression of downstream mediators of T cell activation and mTOR signalling.

### Rapamycin does not synergise with atorvastatin to promote downregulation of co-inhibitory receptors by stimulated CD4^+^ and CD8^+^ T cells

**Figure 9:**
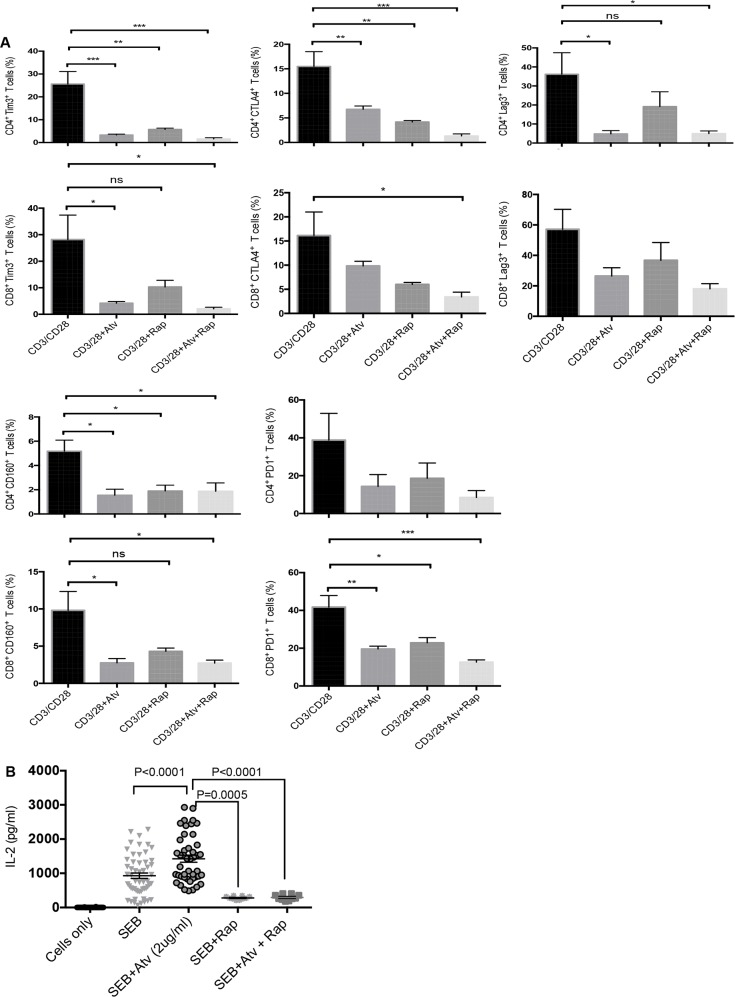
Co-inhibitory receptor expression and IL-2 production in response to atorvastatin and rapamycin treatment **(A)** Bar graphs showing expression of indicated co-inhibitory receptors by CD4^+^ and CD8^+^ T cells stimulated with α-CD3/CD28 and treated with atorvastatin, with or without rapamycin (100nM). **(B)** Scatter plots showing IL-2 production in response to SEB stimulation with or without atorvastatin and/or rapamycin treatment. Data from three independent experiments shown. Bar, mean ± one standard error. P values are defined by ^*^ (P ≤ 0.05) ^**^ (P < 0.01) and ^***^ (P < 0.001).

Since atorvastatin treatment inhibited expression of mTOR by stimulated T cells, we sought to identify whether rapamycin treatment can further increase the downregulation of co-inhibitory receptors observed in response to atorvastatin treatment. Rapamycin treatment during PBMC stimulation with αCD3/CD28 or SEB resulted in reduced expression of co-inhibitory receptors and IL-2 production. Nevertheless, co-treatment with atorvastatin did not have an additive effect on co-inhibitory receptor downregulation when cells were stimulated with aCD3/CD28 (Figure 9A). Similar effects were observed when PBMCs were stimulated with SEB (data not shown). However, the addition of rapamycin significantly reduced IL-2 production by SEB stimulated T cells (Figure 9B). Furthermore, treatment with atorvastatin during SEB-stimulation did not reverse reduced IL-2 production in response to exogenous rapamycin (Figure 9B). Hence, targeting mTOR and concomitant reduction in T cell effector function by rapamycin is not affected by atorvastatin treatment.

### Downregulation of PD-1 expression by atorvastatin correlates with reduced SHP-2 recruitment

**Figure 10:**
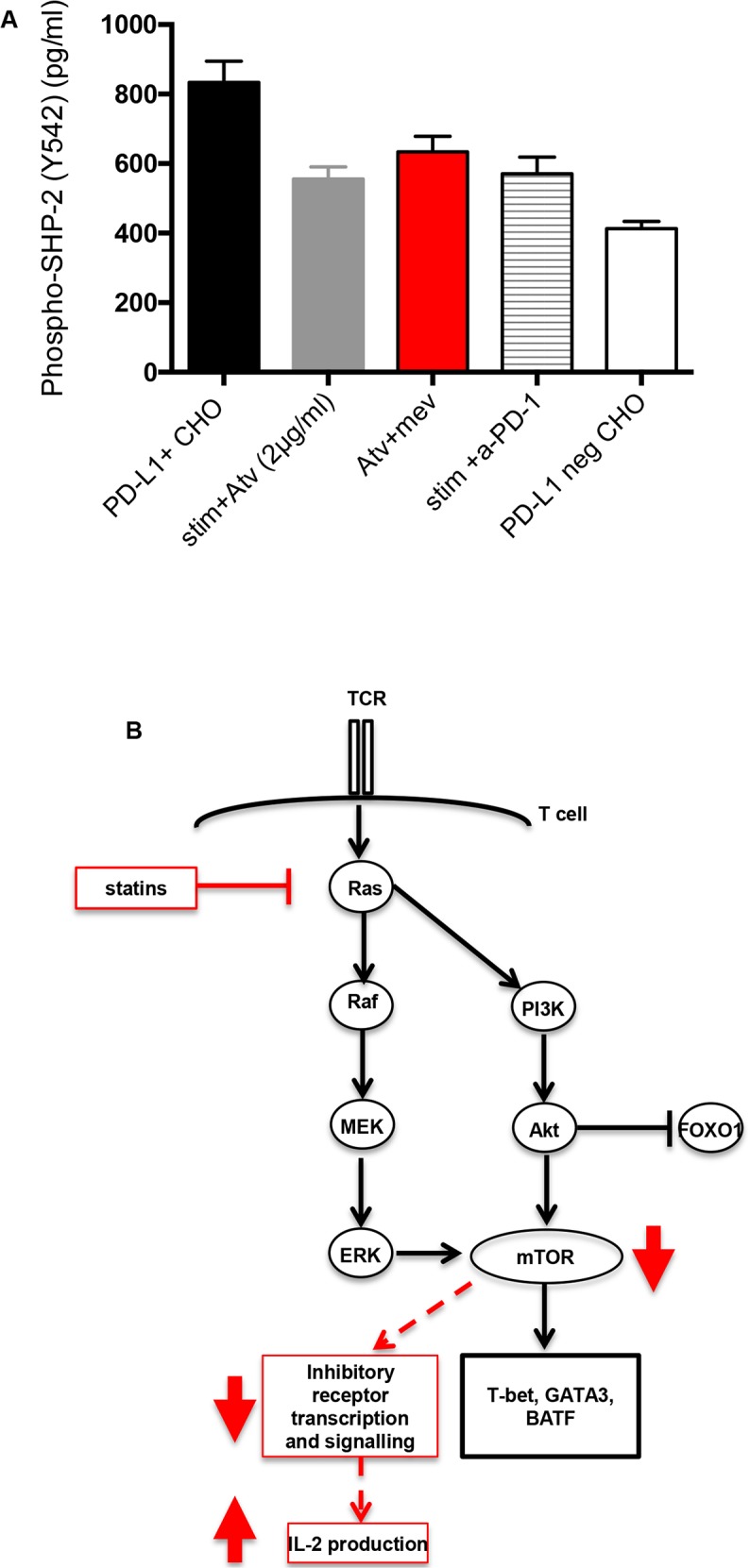
Induction of phospho-SHP-2 in stimulated Jurkat cells **(A)** PD-1-expressing Jurkat T cells were pre-treated with atorvastatin (2 μg/ml) alone, or in combination with L-mevalonate (100μM)) for 48hrs before stimulation with PD-L1-expressing CHO cells. Data showing induction of phospho-SHP-2 in Jurkat T cells after stimulation for 3hrs. Phospho-SHP-2 induction by stimulated Jurkat T cells pre-treated with human α-PD-1 antibody was also determined. PD-L1+ and PD-L1 negative CHO cells were used as positive and negative controls respectively. Bar, mean ± one standard error. **(B)** Proposed mechanism of statin-mediated downregulation of co-inhibitory receptors. Inhibition of Ras-activated Raf/ERK and PI3K/Akt pathways by atorvastatin leads to reduction in mTOR signaling. Akt inhibits FOXO1 signaling. Statin-mediated inhibition mTOR signaling inhibits transcription of T cell effector genes such as T-bet, GATA3 and BATF and possibly co-inhibitory receptors, which are upregulated upon T cell stimulation. Downregulation of co-inhibitory receptors by SEB-stimulated T cells in response to statin treatment corresponds with increased IL-2 production.

We sought to identify the mechanism underpinning atorvastatin-mediated downregulation of co-inhibitory receptors expressed by stimulated CD4^+^ and CD8^+^ T cells. Since addition of atorvastatin to cultures led to reduction in the expression of PD-1 and its ligands PD-L1 and PD-L2 by stimulated T cells and monocytes respectively, we postulated that recruitment of SHP-2, which is triggered upon PD-1-PD-L1 binding will be affected. We found that PD-1-expressing Jurkat cells pre-treated with atorvastatin for 48hrs and stimulated with PD-L1+ CHO cells produced less phosphorylated (Y542) SHP-2 compared to untreated controls (Figure 10A) or Jurkat cells stimulated with PD-L1-negative CHO cells (Figure 10A). Furthermore, we observed similar levels of phospho-SHP2 produced by Jurkat cells treated with anti-PD-1 antibody for 48hrs (Figure 10A). We also investigated whether L-mevalonate treatment could reverse the reduction in phospho-SHP2 induced by atorvastatin. Addition of L-mevalonate in the presence of atorvastatin for 48hrs modestly reversed phospho-SHP2 downregulation compared to atorvastatin-only controls (Figure 10A). Atorvastatin may therefore mediate downregulation of PD-1 expression and subsequent signalling by targeting SHP-2 recruitment and phosphorylation.

## DISCUSSION

The anti-inflammatory properties of statins on T cell function have been demonstrated by their ability to reduce cell activation in response to *in vitro* stimulation and infections [18, 24, 41]. Variations in the immunomodulatory properties of different statins have been reported [16, 18, 42, 43] however, preferential Th2 and regulatory T cell differentiation over pathogenic Th1 and Th17 responses following statin treatment have been noted [44–48].

We have previously shown that atorvastatin inhibits activation of CD4^+^ T cells and ensuing virus replication during HIV-1 infection by restricting the cell cycle and expression of activation markers [24]. In this study, we demonstrate that atorvastatin can also modulate T cell function by promoting downregulation of multiple co-inhibitory receptors. This was dependent on L-mevalonate metabolism as the phenotype was restored by exogenous mevalonate and farnesyl to a lesser extent.

The expression and upregulation of co-inhibitory receptors by healthy human peripheral blood CD4^+^ and CD8^+^ T cells is associated with activation and differentiation status (reviewed in [49, 50]). However, sustained and co-expression of inhibitory receptors are hallmarks of exhausted T cells, which are prominent in chronic viral infections and cancer [2, 3, 32]. The use of statins as preventive and prophylactic measures for cancer therapy have been proposed [51–54] In addition, statins and other lipid-lowering drugs are administered to HIV patients to avert coronary heart disease [55, 56]. In these studies reduction in percentages of TIM-3 and PD-1 by T cells in response to atorvastatin treatment have been reported [18, 25]. In this regard, downregulation of co-inhibitory receptors expression by atorvastatin and other statins may have implications for immunotherapeutic strategies.

Targeting co-inhibitory receptors or immune checkpoints such as PD-1 and CTLA-4 by antibody blockade, alone or in combination, is now an established strategy for treating patients with melanoma and non-small cell lung cancer [57–59]. Moreover, reduction in expression of co-inhibitory receptors in response to antibody blockade has been shown to correlate with restored immune function [7, 60, 61].

In our studies downregulation of co-inhibitory receptors was observed in CD4^+^ and CD8^+^ T cells stimulated with either α-CD3/CD28 or SEB in response to atorvastatin treatment. Irrespective of immunophenotypic variations, we observed significant reduction in the expression of multiple co-inhibitory receptors including PD-1, TIM-3, LAG-3, CTLA-4, CD160, TIGIT and 2B4 in a dose-dependent manner over time. In addition, we observed atorvastatin mediate reduction in co-inhibitory receptor co-expression, which has been demonstrated to indicate more negative regulation of exhausted T cells [3]. We also found that atorvastatin-treatment reduced expression of the co-inhibitory receptor ligands, PD-L1, PDL2 and Gal-9. Statins have been shown to exert their anti-inflammatory effects on monocytes [62–64]. The downregulation in co-expression PDL1 and PDL2 by stimulated CD14^+^CD11b^+^ monocytes indicates that atorvastatin also modulates the function of antigen-presenting cells.

Interestingly, reduction in co-inhibitory receptor expression in response to atorvastatin treatment did not completely correlate with improved immune function, seen in antibody-treated exhausted T cells [7]. This was not unexpected due to obvious differences between *in vitro* stimulation and *in vivo* antigen-specific responses. Furthermore, co-inhibitory receptor expression by α-CD3/CD28- and SEB-stimulated T cells is indicative of T cell activation and differentiation, not exhaustion [49, 50]. However, as we have shown in Figure 1, blockade of co-inhibitory receptors in this system can enhance T cell function. Reduced proliferation exhibited by both α-CD3/CD28- and SEB-stimulated T cells is indicative of statin-mediated inhibition of MAPK and PI3K signalling [14]. Interestingly, in our study atorvastatin treatment did not affect production of IFN-γ. This is in agreement with the report showing negligible effect of simvastatin and atorvastatin on IFN-γ production in a human *in vivo* study [16].

Increased IL-2 production in response to SEB stimulation and atorvastatin treatment is synonymous with restored T cell function in response to blockade of co-inhibitory receptors by antibodies. Nevertheless, reduction in IL-2 production occurs in response to statin-mediated inhibition of MAPK and PI3K signalling [14]. It is likely that a compensatory pathway for IL-2 production is uncoupled or augmented in response to atorvastatin inhibition of MAPK and PI3K-Akt signalling. A study has shown that the inducible T cell kinase (ITK) is required for IL-2 production by SEB-stimulated T cells *in vitro* [65]. In addition ITK signalling has been shown to promote Th2 differentiation [66, 67], which can also be increased by statin treatment [20, 45, 68]. We found that expression of the Th2 master transcription factor, GATA3 [69] by SEB-stimulated and atorvastatin-treated T cells was reduced compared to stimulated-only controls. This is probably due to fact that GATA3 expressed in response to SEB stimulation is due to TCR signalling [70] and not Th2 differentiation. However, our observations show that atorvastatin treatment has the potential to mediate increased IL-2 production and restoration of immune function.

Our results suggest that atorvastatin modulates T cell function by reducing expression of multiple co-inhibitory receptors. So we sought to identify whether there exists a common downstream target and an associated negative feedback mechanism utilised by atorvastatin that impacts co-inhibitory receptor expression. We found expression of mTOR, which signals downstream of Ras-activated PI3K-Akt and Ras-ERK pathways and promotes effector T cell function [37–39, 71] was significantly downregulated in atorvastatin-treated and stimulated T cells. mTOR is involved in the activation of several downstream effector pathways including immune receptor signalling, cell trafficking and metabolism [72]. Multiple co-inhibitory receptors are variably upregulated by effector T cells in response to T cell stimulation [49, 50]. It is likely that inhibition of Ras and subsequent mTOR signalling by atorvastatin during T cell stimulation compromises subsequent co-inhibitory receptor transcription. In addition, reduction in expression of other downstream effector T cell genes such as T-bet, BATF, GATA3 and FOXO1 by atorvastatin (Figure 8, Figure 10B) may amplify this response.

Activated PD-1 and CTLA-4 associate with the protein tyrosine phosphatase SHP-2 resulting in downregulation of TCR signalling and the PI3K pathway [73–76]. Our results show that atorvastatin inhibits phosphorylation of SHP-2 at levels comparable to anti-PD-1 antibody (Figure 10A). From this observation, we can deduce that inhibition of PD-1 and CTLA-4 by atorvastatin interferes with ligand binding and downstream signalling events. It will be interesting to identify whether atorvastatin interacts with and influences signalling of other co-inhibitory receptors in a similar manner. It has been shown that upon α-CD3/CD28-stimulation, TIM-3 sequesters Lck and PLC-γ, thereby inhibiting TCR signalling [77]. It has also been suggested that TIM-3 indirectly targets PI3K-Akt signalling [78]; a potential mechanism for atorvastatin modulation.

Taken together, here we have demonstrated that atorvastatin mediates gross downregulation of co-inhibitory receptors upon T cell stimulation. This activity appears to be specific and selective for some molecules such as inhibitory receptors. In agreement, we have previously shown that atorvastatin downregulates CCR5 but not CXCR4 [24] and here we have shown that atorvastatin upregulates expression of CD25 on CD4^+^ T cells in a dose dependent manner (Supplementary Figure 6A). This represents yet another mechanism by which atorvastatin and potentially other statins control immune activation. Additionally, these atorvastatin activities have public health implications due to the high percentage of individuals that use statins on a daily basis [79]. Atorvastatin-induced reduction of co-inhibitory receptor expression may be beneficial in chronic settings to enhance restoration of T cell function. In this regard, in depth studies are required to understand how such reduction in co-inhibitory receptor expression impacts tumour growth, viral load, T cell function and the proliferative burst [80]. Since, monotherapy may be relatively ineffective in majority of cancer patients, combination therapy of immune checkpoint inhibitors is under expensive consideration. Therefore, the broad-spectrum effects of atorvastatin on down regulation of multiple co-inhibitory receptors is an interesting property that merits further *in vivo* analysis.

Furthermore, active form of lipophilic statins such as atorvastatin has been detected in other tissues including brain [81], suggesting lipophilic statins have an advantage to penetrate in other tissues. The essential role of mevalonate pathway in many cancers, coupled with safer drugs that can circulate into extra-hepatic tissues may provide an appealing rational for further investigating of their metabolic and immunological pathways in cancer. Thus, population studies to identify if atorvastatin and other stains usage deters the onset or progression of chronic disease will be useful in establishing it as a prophylactic measure against chronic conditions associated with T cell exhaustion.

## MATERIALS AND METHODS

### Study population

PBMC samples from > 30 HIV, HCV and HBV seronegative individuals were used for these studies. The appropriate Institutional Review Boards at the University of Alberta approved the studies, IRB #Pro00046064. All study participants gave written informed consent to participate in this study. All studies were performed in accordance with the relevant guidelines and regulations.

### Cell isolation

Whole blood was processed by density gradient centrifugation using Ficoll-Paque PREMIUM (GE Healthcare). Peripheral blood mononuclear cells (PBMCs) were collected and washed twice with pre-warmed RPMI-1640 medium (Sigma-Aldrich) containing 10% fetal bovine serum (FBS), L-gutamine and antibiotics. The cells were cryopreserved in FBS containing 10% DMSO (Sigma-Aldrich) in liquid nitrogen until use. In some cases, fresh PBMCs were used for cell culture. We did not observe any difference between fresh versus frozen PBMCs.

### Cell culture and reagents

2.5 × 10^5^ PBMCs per well were plated in a 96 U-bottomed plate and stimulated with 100ng/ml SEB (Sigma) or soluble α-CD3 (1.5μg/ml) and α-CD28 (0.5μg/ml; BD Biosciences) for 24, 48 or 72 hrs at 37°C, 5% CO_2_ in the presence or absence of atorvastatin (0.5 μg/ml, 1 μg/ml or 2 μg/ml; Sigma-Aldrich). For some experiments PBMCs were stimulated in the presence of cholesterol (50μM, 100μM and 200μM; Sigma-Aldrich), L-mevalonate (lithium salt, 100μM; Sigma-Aldrich), farnesyl (5μM, Sigma-Aldrich) or rapamycin (25nM, 50nM and 100nM; Sigma-Aldrich) with atorvastatin. Unstimulated and untreated PBMCs were used as negative controls.

### Flow cytometry

PBMCs were stained with the following surface and intracellular antibodies: LIVE/DEAD® Fixable Aqua Dead Cell Stain Kit, (ThermoFisher Scientific), to exclude dead cells, CD3 (SK7), CD4 (RPA-T4), CD8 (SK1 and RPA-T8), CTLA-4 (BNI3), TIM-3 (F38-2E2), CD244 (C1.7), CD71 (M-A712), LAG-3 (3DS223H), CD160 (BY55), PD-1 (EH12.1), PDL-1 (M1H1), PDL-2 (MIH18), CD11b (ICRF44), CD14 (M5E2) and Gal-9 (9M1-3) (BD Biosciences), TIGIT (MBSA43, eBioscience). Following staining cells were fixed with 4% paraformaldehyde, before analysis. Cells were acquired using an LSR FortessaSORP flow cytometer (BD Biosciences) and analysed with FlowJo software (Ashland, Oregon, USA). For CFSE labelling, PBMCs were labelled with 1.25μM CFSE (ThermoFisher Scientific) as previously described [82] before stimulation with SEB or a-CD3/CD28.

### ELISA

After PBMC stimulation, cell culture supernatants were harvested and used to measure concentrations of IL-2, IFN-γ and TNF-α by ELISA (Human DuoSet ELISA kits, R&D Systems) according to manufacturer's instructions.

### T cell isolation

T cells from stimulated PBMCs were isolated using the EasySep™ Human T cell isolation kit (Stemcell™ Technologies) according manufacturer's instructions. The purity of isolated T cells was determined by flow cytometry (approximately 95%, Supplementary Figure 6B).

### RNA isolation, cDNA synthesis and RT-PCR

RNA was isolated from approximately 1 × 10^6^ T cells using the RNAeasy mini kit (Qiagen). The concentration of isolated RNA samples was determined using a nanodrop spectrophotometer (ThermoFisher Scientific). Only samples with 260/280 ratios between 1.8 and 2.0 were used for further analysis. For cDNA synthesis, 100ng RNA was reverse transcribed using the miScript II RT Kit (Qiagen) and the T100 Thermal Cycler (BIO-RAD). Real-time PCR (RT-PCR) was performed using the CFX96 Touch™ Real-Time PCR Detection System (BIO-RAD). Quantitect Primer Assays (Qiagen) for the following genes were carried out: *MTOR, TBX21, GATA3, FOXO1* and *BATF*. β2-microglobulin (*B2M*) was used as an internal control. Data was analysed using the 2^-ΔΔCT^ method.

### Jurkat T cell stimulation and Phospho-SHP-2 (Y542) ELISA

Recombinant Jurkat T cells that constitutively express PD-1 (BPS Bioscience) were pre-treated with atorvastatin only (2 μg/ml), or with L-mevalonate (100μM) for 48hrs. For some experiments, Jurkat T cells were pre-treated with human α-PD-1 antibody (pembrolizumab, 5 μg/ml) for 48hrs before stimulation. Treated Jurkat T cells were co-cultured with recombinant Chinese Hamster Ovary (CHO-K1) cells constitutively expressing human PD-L1 and an engineered T cell receptor (BPS Bioscience) at a ratio of 10:1 for 3hrs at 37°C. Jurkat cell lysates were collected and phosphorylation of the Src homology region 2-domain phosphatase 2 (SHP-2) was measured using sandwich ELISA (R&D Systems) according to manufacturer's instructions.

### Statistical analysis

Statistical analyses were performed using the Mann–Whitney non-parametric test for paired groups and the Kruskal-Wallis test for un-matched groups. One-way ANOVA was used to analyse more than two groups. P-values less than 0.05 were considered statistically significant.

## SUPPLEMENTARY MATERIALS FIGURES


