# Supplementary material for: Atorvastatin downregulates co-inhibitory receptor expression by targeting Ras-activated mTOR signalling
Source: Oncotarget. 2017 Sep 18;8(58):98215–32. doi: 10.18632/oncotarget.21003 (PMC5716724; doi:10.18632/oncotarget.21003)
Supplement: Supplementary file 1 [file oncotarget-08-98215-s001.pdf]

Atorvastatin downregulates co-inhibitory receptor expression by targeting Ras-activated mTOR signalling

SUPPLEMENTARY MATERIALS

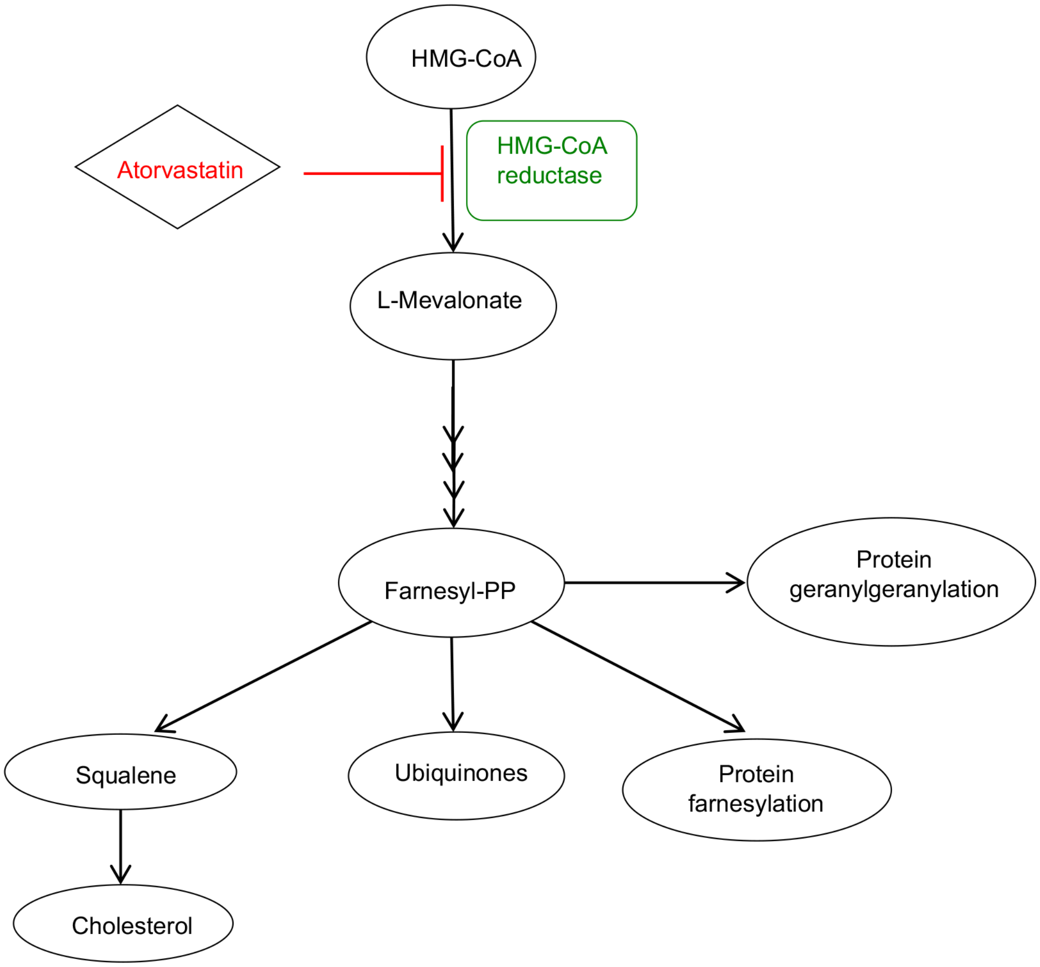

Supplementary Figure 1: Schematic representation of L-Mevalonate metabolism and downstream metabolites. Hydroxy-metyl glutaryl Coenzyme A reductase (HMG-CoA reductase), the target enzyme for statins like Atorvastatin, converts HMG-CoA into L-Mevalonate. L-Mevalonate is the main substrate of downstream biochemical compounds (cholesterol, ubiquinones) with essential role in biological activities of the cells. Farnesyl pyrophosphate (Farnesyl-PP) downstream of L-mevalonate influences the induction of multiple molecules as shown.

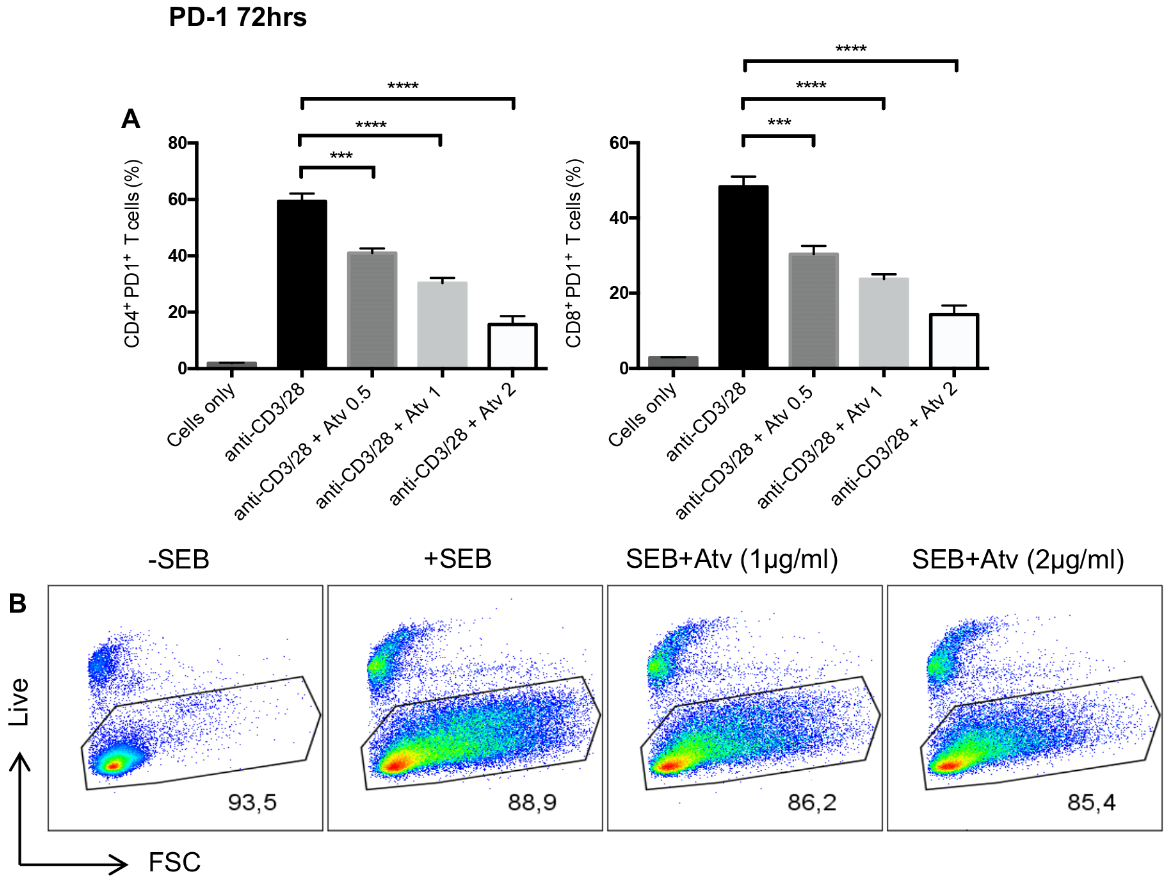

Supplementary Figure 2: Expression of co-inhibitory receptors by stimulated T cells in response to atorvastatin treatment. **(A)** Bar graphs showing cumulative expression of PD-1 by CD4+ (left) and CD8+ (right) T cells after α-CD3/CD28 stimulation for 72hrs with increasing doses of atorvastatin (0.5, 1 and 2μg/ml). **(B)** Representative dot plots showing percentage of live cells from unstimulated, SEB-stimulated and SEB-stimulated plus atorvastatin-treated cultures. P values are defined by *** ($P < 0.001$) and **** ($P < 0.0001$).

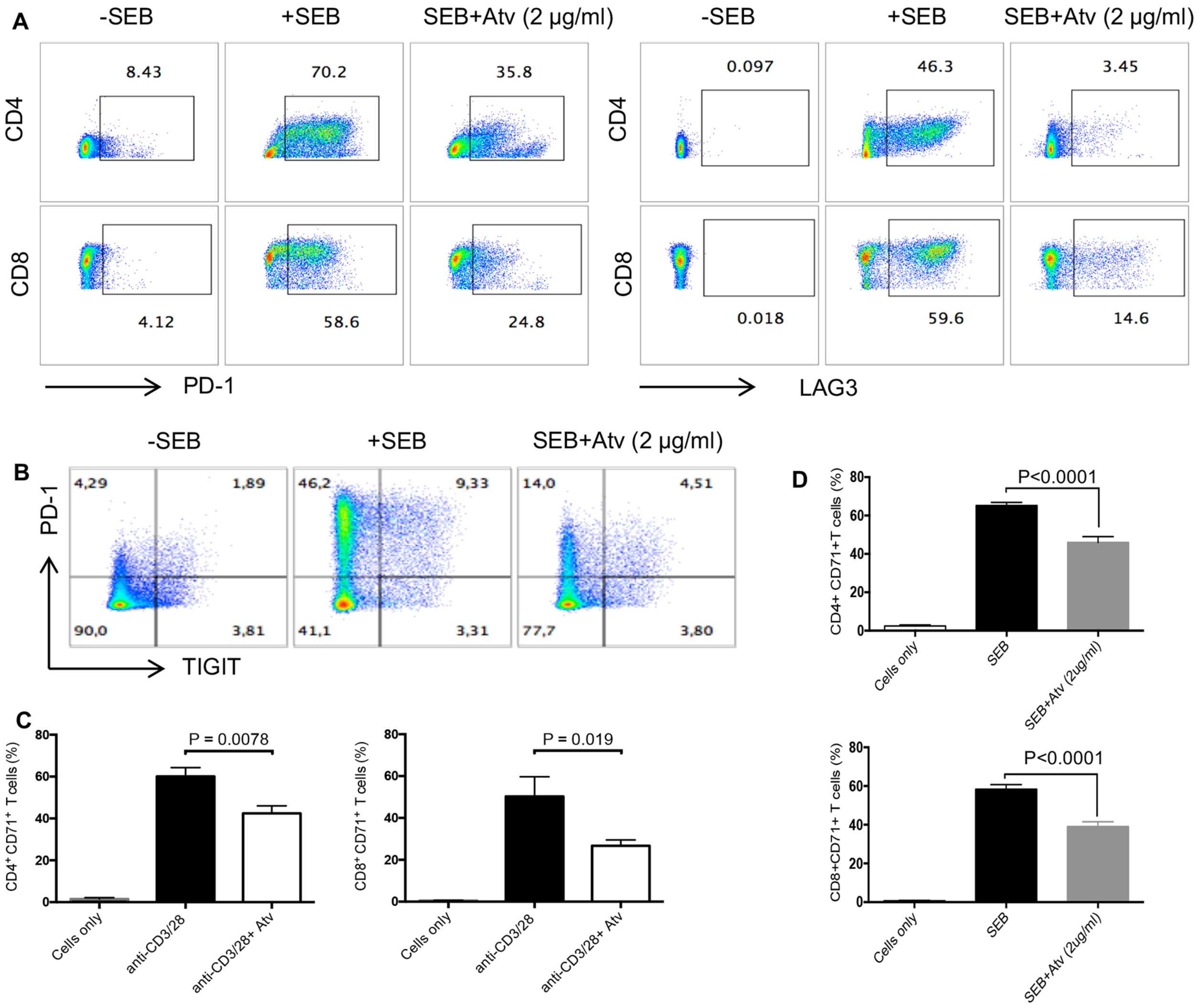

Supplementary Figure 3: (A) Representative dot plots showing expression of PD-1 and LAG-3 or **(B)** co-expression of TIGIT and PD-1 by CD4[+] and CD8[+] T cells in response to SEB stimulation with or without atorvastatin (2 µg/ml) after 72hrs. **(C)** Expression of CD71 by CD4[+] and CD8[+] T cells stimulated with α-CD3/CD28. **(D)** Expression of CD71 by CD4[+] and CD8[+] T cells stimulated with SEB for 48hrs. Data from three to five independent experiments are shown. Bar, mean ± one standard error.

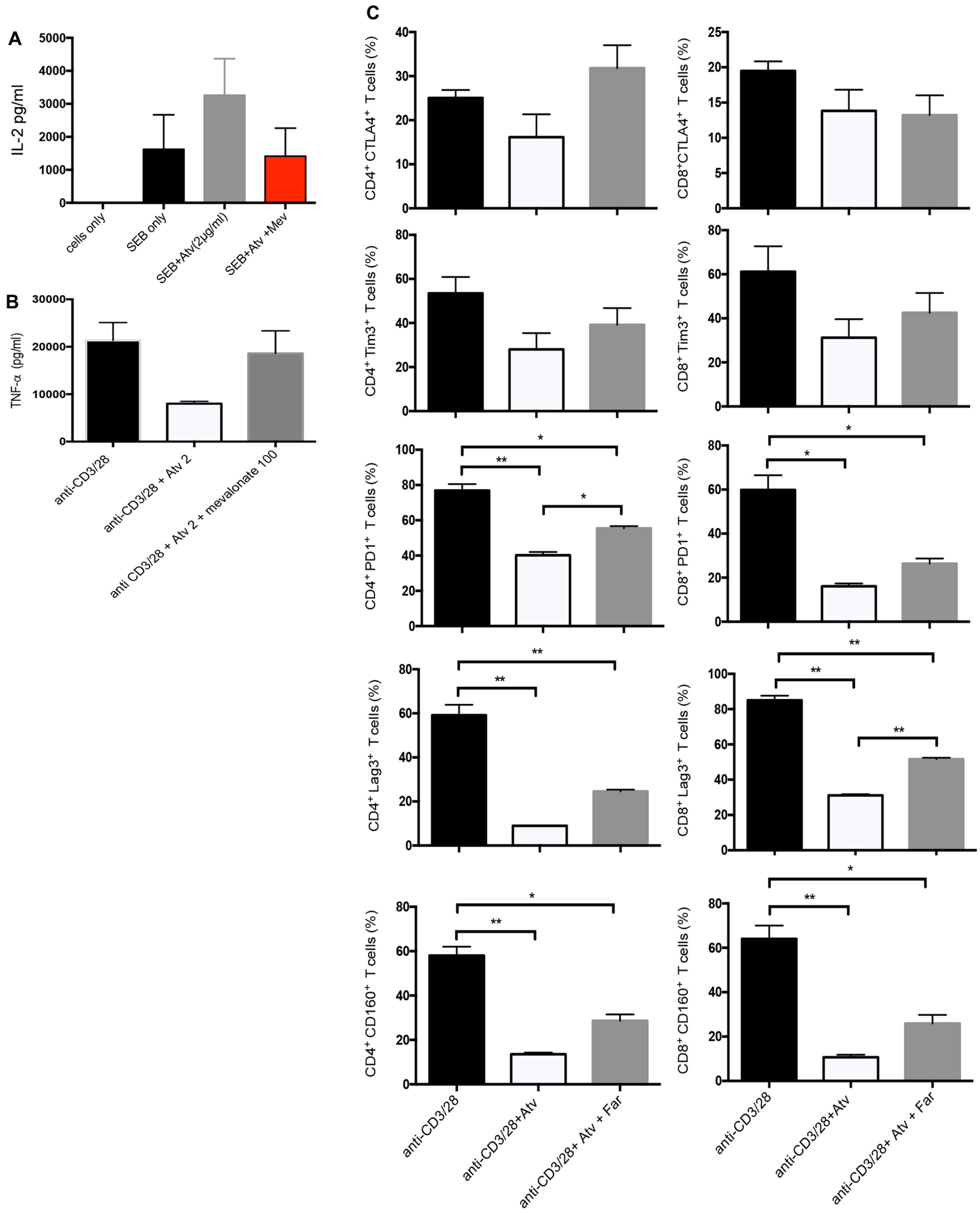

Supplementary Figure 4: Effect of L-mevalonate and farnesyl on atorvastatin-treated cells. **(A)** Concentration of IL-2 in cell culture supernatants from SEB-stimulated PBMCs treated with atorvastatin with or without L-mevalonate for 48 hrs. **(B)** TNF-α produced in response to α-CD3/CD28-stimulation (48hrs) in the presence of atorvastatin only or in combination with L-mevalonate. **(C)** Expression of co-inhibitory receptors by CD4+ and CD8+ T cells stimulated with α-CD3/CD28 with atorvastatin alone or in combination with farnesyl (5µM) for 48hrs.

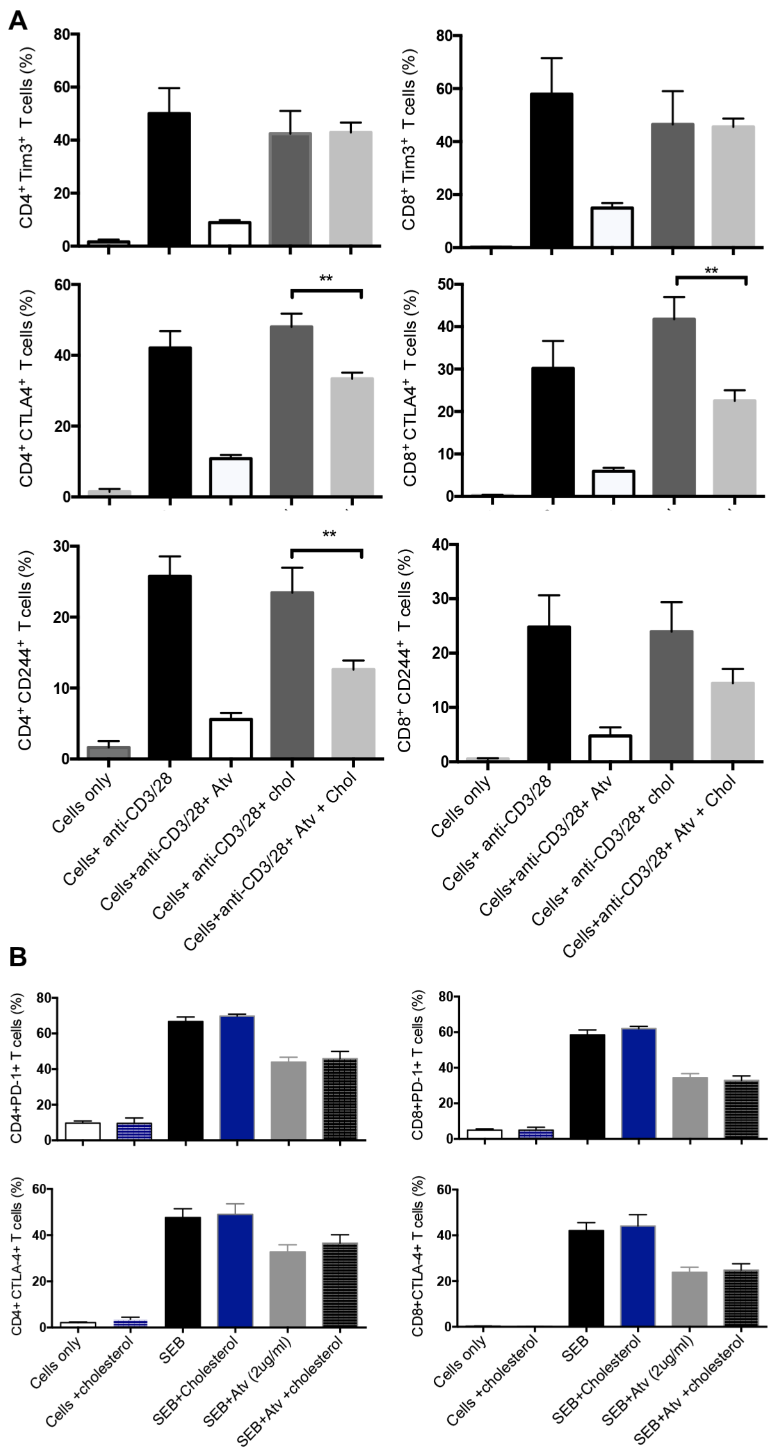

Supplementary Figure 5: Effect of cholesterol on atorvastatin-treated cells. **(A)** Expression of co-inhibitory receptors by $CD4^+$ and $CD8^+$ T cells stimulated with α-CD3/CD28 with atorvastatin alone or in combination with cholesterol (200μM) for 48hrs. **(B)** Co-inhibitory receptor expression by SEB-stimulated $CD4^+$ and $CD8^+$ T cells treated with atorvastatin, alone or in combination with cholesterol for 72hrs. Data from three independent experiments are shown. Bar, mean ± one standard error. P values are defined by * ($P \leq 0.05$) and ** ($P < 0.01$).

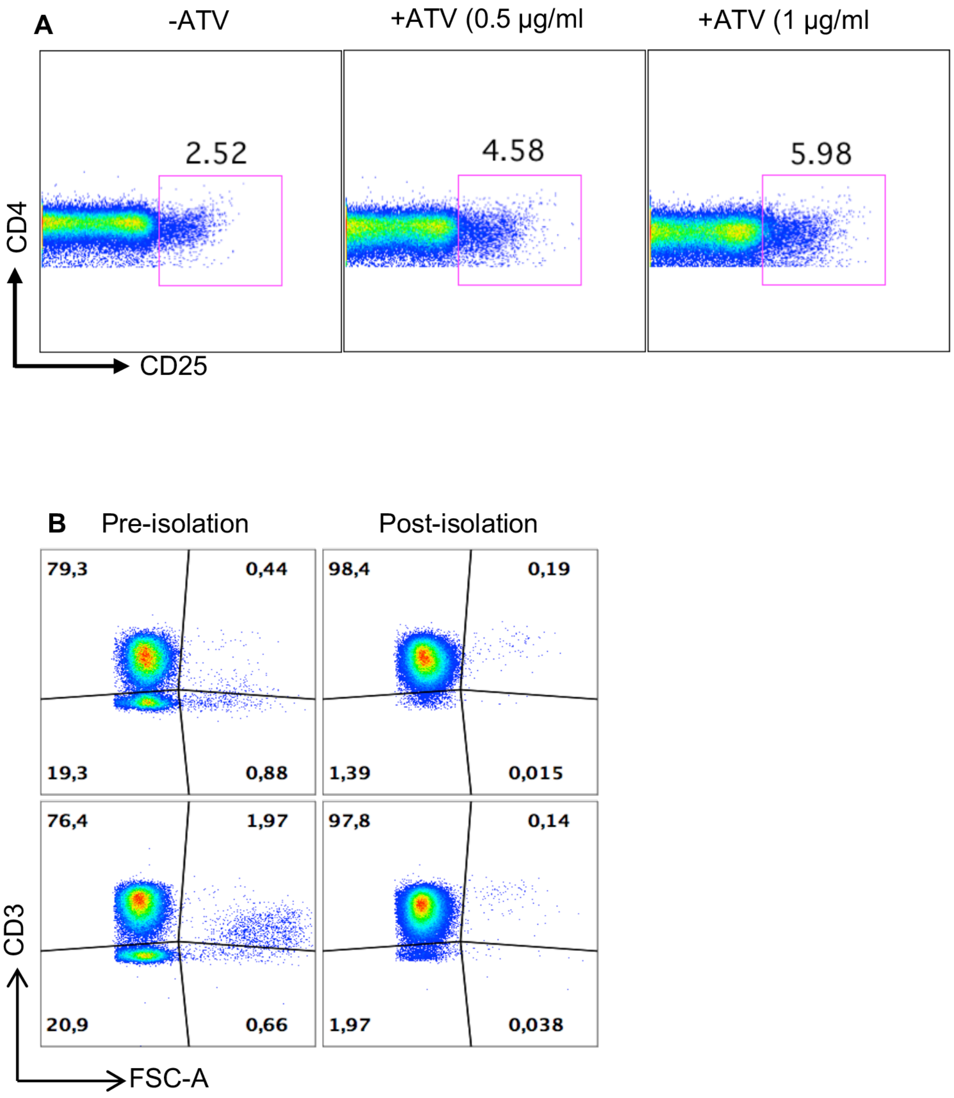

Supplementary Figure 6: (A) Representative plots showing expression of CD25 on CD4 T cells in the presence of atorvastatin. **(B)** Two representative dot plots showing purity of CD3+ T cells pre- and post-T cell isolation using immunomagnetic negative selection.
